# Identification and characterization of a new true lipase isolated through metagenomic approach

**DOI:** 10.1186/1475-2859-10-54

**Published:** 2011-07-15

**Authors:** Arnaldo Glogauer, Viviane P Martini, Helisson Faoro, Gustavo H Couto, Marcelo Müller-Santos, Rose A Monteiro, David A Mitchell, Emanuel M de Souza, Fabio O Pedrosa, Nadia Krieger

**Affiliations:** 1 Department of Biochemistry and Molecular Biology, Federal University of Paraná, Curitiba/PR, Brazil; 2 Department of Chemistry, Federal University of Paraná, Curitiba/PR, Brazil

## Abstract

**Background:**

Metagenomics, the application of molecular genomics to consortia of non-cultivated microbes, has the potential to have a substantial impact on the search for novel industrial enzymes such as esterases (carboxyl ester hydrolases, EC 3.1.1.1) and lipases (triacylglycerol lipases, EC 3.1.1.3). In the current work, a novel lipase gene was identified from a fosmid metagenomic library constructed with the "prokaryotic-enriched" DNA from a fat-contaminated soil collected from a wastewater treatment plant.

**Results:**

In preliminary screening on agar containing 1% tributyrin, 2661 of the approximately 500,000 clones in the metagenomic library showed activity. Of these, 127 showed activity on agar containing 1% tricaprylin, while 32 were shown to be true lipase producers through screening on agar containing 1% triolein. The clone with the largest halo was further characterized. Its lipase gene showed 72% identity to a putative lipase of *Yersinia enterocolitica *subsp. *palearctica *Y11. The lipase, named LipC12, belongs to family I.1 of bacterial lipases, has a chaperone-independent folding, does not possess disulfide bridges and is calcium ion dependent. It is stable from pH 6 to 11 and has activity from pH 4.5 to 10, with higher activities at alkaline pH values. LipC12 is stable up to 3.7 M NaCl and from 20 to 50°C, with maximum activity at 30°C over a 1 h incubation. The pure enzyme has specific activities of 1722 U/mg and 1767 U/mg against olive oil and pig fat, respectively. Moreover, it is highly stable in organic solvents at 15% and 30% (v/v).

**Conclusions:**

The combination of the use of a fat-contaminated soil, enrichment of prokaryotic DNA and a three-step screening strategy led to a high number of lipase-producing clones in the metagenomic library. The most notable properties of the new lipase that was isolated and characterized were a high specific activity against long chain triacylglycerols, activity and stability over a wide range of pH values, good thermal stability and stability in water-miscible organic solvents and at high salt concentrations. These characteristics suggest that this lipase has potential to perform well in biocatalytic processes, such as for hydrolysis and synthesis reactions involving long-chain triacylglycerols and fatty acid esters.

## Background

Lipases (triacylglycerol lipases, EC 3.1.1.3) are enzymes that act on ester bonds, either hydrolyzing or synthesizing them, depending on the amount of water in the reaction medium [1-3]. True lipases attack triacylglycerols that contain long-chain fatty acids. They are important biocatalysts for various biotechnological applications due to their useful features, such as stability in organic solvents, broad substrate specificity, stereoselectivity and regioselectivity [4].

Although lipases can be obtained from plants and animals, microbial lipases possess useful features such as high yield and low production cost, diversity in catalytic activities, amenability to genetic manipulation, stability in organic solvents and broad substrate specificity [5]. While a large number of different lipases have been discovered and commercialized [4], new lipases with better characteristics are desirable, such as high activity and stability in non-aqueous media, which are useful for biodiesel production through transesterification [6-8], regiospecificity for the modification of oils and fats to produce specific-structured lipids [9,10] and stability under alkaline conditions and in the presence of surfactants for use in detergent formulations [11].

The vast majority of lipases that are currently used industrially were isolated from cultivated microbes [12], however, in more recent times, it has become difficult to obtain truly different lipases by this method. To overcome this limitation, molecular biology and protein engineering strategies have been used, such as directed evolution [13], rational design [14,15] and metagenomics [16-20].

The metagenomics approach, the application of molecular genomics to consortia of non-cultivated microbes, has a substantial impact on the search for novel industrial enzymes due to the vast diversity of genetic material analyzed [21-24]. The advantage of metagenomics can be summarized in numerical terms since more than 99% of microorganisms are not amenable to cultivation [25], either due to their required growth conditions being unknown or to their need to grow within a microbial consortium.

Although various esterases and so-called lipases have been isolated through the metagenomic approach over the last decade [26-31], relatively few papers report true lipases [32-37]. Several of the enzymes obtained have activity against medium-chain triacylglycerols, long-chain monoacyl glycerols or long-chain nitrophenyl acyl esters, but do not have activity against triacylglycerols that contain long-chain fatty acids, such as vegetable oils or animal fats. For example, the so-called PLP and Est1 lipases, both of which were isolated through the metagenomic approach [38], and *Sulfolobus acidocaldarius *lipase [39] are active against *p*-nitrophenyl palmitate (*p*NPP) but not against trioleoylglycerol. As a result, these so-called lipases simply cannot be considered as true lipases.

As new lipases with high activity against oils and fats have industrial potential, in the current work we used three strategies in combination in an attempt to improve the chances of obtaining a true lipase through the metagenomic approach, namely (i) a high-fat soil as the original source of the DNA, (ii) a prokaryotic DNA enrichment step and (iii) a three-stage screening strategy in which the third stage tested for true lipase activity.

## Results

### Sample collection, metagenomic library construction and activity screening

Samples were collected from the bank of an anaerobic lagoon of the wastewater treatment plant of a meat packing and dairy industry. The effluent from the plant has a high animal fat content that contaminates the soil surrounding the lagoon, presumably favoring the development of a microbiota capable of degrading fats. Consequently, this soil sample should be enriched with lipase genes.

**Figure 1:**
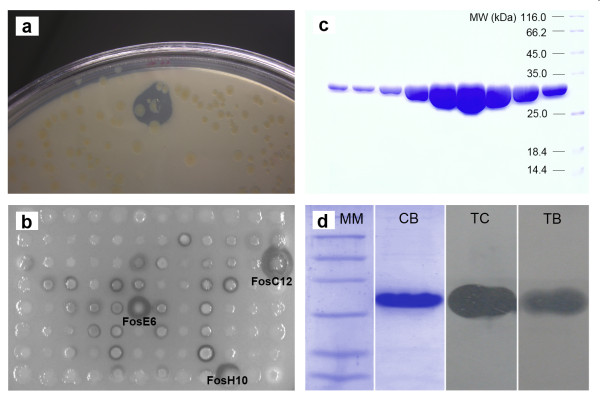
**Isolation, purification and identification of LipC12**. (a) Identification of a lipolytic clone that formed a hydrolysis halo in the tributyrin plate assay. (b) Hydrolysis halos formed by lipase-producing clones in the triolein plate assay. FosC12, FosE6 and FosH10 showed the largest hydrolysis halos and were chosen for sequencing. (c) SDS-PAGE of fractions of His-tagged LipC12 eluted from the affinity chromatography column. (d) SDS-PAGE and zymogram analyses of LipC12. The lanes correspond to molecular marker (MM), coomassie blue staining (CB) and lipolytic activity of LipC12 on the SDS-PAGE gel, using tricaprylin (TC) and tributyrin (TB) as substrates.

The generated metagenomic library has approximately 500,000 clones. The screening strategy used involved the use of LB agar with two or more different types of triacylglycerols to differentiate lipases from esterases [19,40]. Thus, the 2,661 clones that showed activity against tributyrin (Figure 1a) were screened on LB agar containing 1% (v/v) tricaprylin and 127 clones with activity were selected. In order to identify true-lipase producers, these clones were screened on LB agar containing 1% (v/v) triolein, resulting in 32 with activity. Three clones, FosC12, FosE6 and FosH10, which showed the largest hydrolysis halos on triolein (Figure 1b), were selected for further characterization.

### Sequencing of LipC12 and preliminary sequence analysis

The fosmids FosC12, FosE6 and FosH10 were fragmented by nebulization and subcloned into the pUC18 vector, producing three subclone libraries. The inserts of subclones that expressed lipolytic activity on tributyrin agar plates were fully sequenced and sequence comparison revealed that all three fosmids contained the same lipase gene (result not shown). The lipase gene from fosmid FosC12, denominated *lipC12*, was selected for further characterization. Amino acid sequence alignment showed that *lipC12 *codes for a lipase having 72% identity to the putative lipase of *Yersinia enterocolitica *subsp. *palearctica *Y11 [GenBank:CBY26912]. Neither transmembrane domains nor a signal peptide were identified and LipC12 was predicted to be a soluble protein. No chaperone sequence gene located close to the *lipC12 *gene was found in the *lipC12 *contig, suggesting that LipC12 has a chaperone-independent folding.

The *lipC12 *gene was amplified and cloned into pET-28a(+) and transformed into *E. coli *BL21(DE3) cells to express the N-terminal (His)6-tagged protein. No mutation occurred in the *lipC12 *sequence in the course of these cloning procedures.

### Overexpression and purification of LipC12 lipase

The overexpressed LipC12 has a molecular weight of 33 395.8 Da and was in the soluble fraction, as judged by SDS-PAGE (result not shown). BL21(DE3) cells carrying the pET28a-*lipC12 *plasmid were induced at 20°C for 16 h to maximize the protein yield. LipC12 was purified in one step using a HiTrap Chelating HP affinity column, as shown by SDS-PAGE of eluted fractions (Figure 1c). The eluted fractions containing LipC12 were combined and dialyzed against Tris-HCl buffer to concentrate the protein solution and eliminate imidazol. Glycerol at 50% (w/v) final concentration was added to the protein storage buffer as a cryoprotective agent. The enzyme preparation (5.78 μg/μL) was more than 97% pure as judged by densitometric analysis of SDS-PAGE.

### Zymogram and mass spectrometry analysis

Zymographic analysis was carried out using tributyrin and tricaprylin as substrates. The clear bands that were obtained in the 30 kDa region using both substrates reveal that the purified enzyme was active, with a larger clearing zone occurring in the tricaprylin gel than in the tributyrin gel (Figure 1d). MALDI-TOF/MS confirmed that the purified enzyme is LipC12, with 68.4% of sequence coverage (result not shown). Together, these results confirm the identity of the purified protein.

### Protein sequence analysis

**Figure 2:**
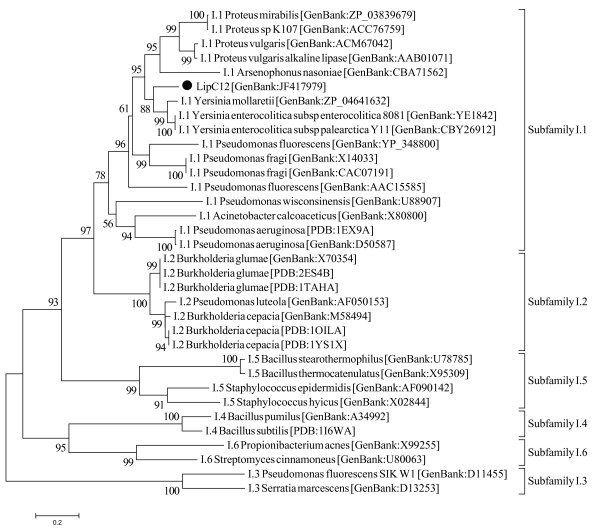
**Phylogenetic analysis of LipC12 and closely related proteins**. The enzymes most similar to LipC12 are those from Enterobacteria, such as *Yersinia sp*., *Proteus sp*. and *Arsenophonus sp*. LipC12 is a member of subfamily I.1 [42], with closest homology to *Yersinia sp*. lipases. Except for LipC12, the protein sequences were retrieved from GenBank (NCBI). The phylogenetic tree was generated using MEGA 5 [65]. The scale represents the number of amino acid substitutions per site.

LipC12 has 293 amino acids and an identity of 72% with the putative lipases of *Yersinia enterocolitica *subsp. *palearctica *Y11 [GenBank:CBY26912] and of *Yersinia enterocolitica *subsp. *enterocolitica *8081 [GenBank:YE1842]. The domain analysis carried out using Pfam showed that the enzyme probably has a type I α/β-hydrolase fold [41] between residues 23 and 227. Phylogenetic analysis showed that the most similar enzymes to LipC12 are those from enterobacteria, such as *Yersinia sp*., *Proteus sp*. and *Arsenophonus sp*. (Figure 2). Among the lipases that have known 3D structure, LipC12 possesses 47%, 41% and 41% of identity with lipases of *Pseudomonas aeruginosa *[PDB:1EX9A], *Burkholderia glumae *[PDB:2ES4B] and *Burkholderia cepacia *[PDB:1OILA], respectively.

**Figure 3:**
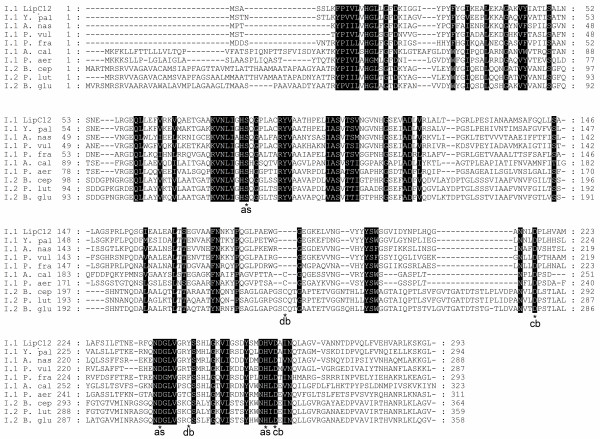
**Characteristic residues in multiple sequence alignment between LipC12 and lipases from subfamilies I.1 and I.2**. Conserved residues of the active site (as), cysteine residues forming the disulfide bridge (db) and aspartic residues involved in the calcium binding domains (cb) are annotated. Shaded regions indicate similar amino acids in all the aligned sequences. The accession number and the organism name of the aligned sequences are (from top to the bottom): [GenBank:JF417979], LipC12 lipase; [GenBank:CBY26912], lipase from *Yersinia enterocolitica *subsp. *palearctica *Y11; [GenBank:CBA71562], lipase from *Arsenophonus nasoniae*; [GenBank:ACM67042], lipase from *Proteus vulgaris*; [GenBank:CAC07191], lipase from *Pseudomonas fragi*; [GenBank:X80800], lipase from *Acinetobacter calcoaceticus*; [GenBank:D50587], lipase from *Pseudomonas aeruginosa*; [GenBank:M58494], lipase from *Burkholderia cepacia*; [GenBank:AF050153], lipase from *Pseudomonas luteola*; [GenBank:X70354], lipase from *Burkholderia glumae*. The figure was generated with GeneDoc 2.7 software.

The LipC12 catalytic triad is predicted to be formed by Ser^83^, Asp^238 ^and His^260 ^(Figure 3) and the nucleophilic Ser^83 ^residue appears in the conserved pentapeptide Gly-X-Ser-X-Gly [41]. Two Asp residues of LipC12 (Asp^217 ^and Asp^262^) form a calcium binding pocket that is conserved in lipases of subfamilies I.1 and I.2 [42,43]. LipC12 contains only one Cys residue, similar to the lipases of *Pseudomonas fragi *[GenBank:CAC07191], *Proteus vulgaris *[GenBank:AAB01071, GenBank:ACM67042], *Yersinia enterocolitica *[GenBank:CBY26912, GenBank:YE1842] and *Yersinia mollaretti *[GenBank:ZP_04641632], and therefore does not form a disulfide bridge as found in the lipases of *Pseudomonas aeruginosa *[PDB:1EX9A], *Burkholderia glumae *[PDB:2ES4B] and *Burkholderia cepacia *[PDB:1OILA]. The expression of lipases belonging to subfamilies I.1 and I.2 in an active form often depends on a chaperone protein named lipase-specific foldase, Lif, that is usually encoded in an operon with its cognate lipase [1,42]. This chaperone is absent in the LipC12 operon and has not yet been found or described for the lipases of subfamily I.1 that have the highest identities with LipC12, as is the case of *Yersinia enterocolitica *[GenBank:CBY26912, GenBank:YE1842], *Yersinia mollaretti *[GenBank:ZP_04641632], *Proteus vulgaris *[GenBank:AAB01071, GenBank:ACM67042] and *Pseudomonas fragi *[GenBank:CAC07191] lipases. Thus, it is possible to conclude that LipC12 belongs to subfamily I.1.

### Spectrophotometric determinations of lipase activity using *p*NP substrates

#### Effect of chelating agents and metal ions on LipC12 activity

LipC12 activity decreases in the presence of the chelating agents EDTA and EGTA, more intensely so for EGTA, which preferentially binds calcium (Table 1). This result suggested that calcium might be a preferred cofactor of LipC12. To check whether LipC12 has a preference for binding Ca^2+^, an experiment was done in which the metal was depleted by EDTA prior to the testing of the activity in the presence of excess metal. Maximum activity was achieved with the addition of Ca^2+^, but activity was also restored at lower levels in the presence of other divalent cations, such as Cu^2+^, Co^2+^, Mn^2+ ^and Ni^2+^. This result, when taken together with the presence of a putative calcium binding pocket, is strong evidence for LipC12 being calcium ion dependent. In experiments undertaken without a prior chelation step, in which the calcium binding pocket was presumably occupied by a calcium ion, the presence of 1 mM of monovalent cations (Rb^+^, K^+^, Cs^+^, Na^+ ^or Li^+^) enhanced LipC12 activity (Table 1). The most likely explanation for this is that the presence of these cations improves the solubility of *p*NPP within the emulsion. Evidence of this increase in solubility is given by the fact that at 1.5 M NaCl the *p*NPP emulsion remained transparent even without heating.

**Table 1:** Effect of some additives, substrate chain length and temperature on the activity of LipC12

Variable	Relative	Variable	Relative	Variable	Relative
	activity (%)		activity (%)		activity (%)
Cation (1 mM) after EDTA chelation^a^		Cation (1 mM) without EDTA chelation		Substrate specificity	
None	< 0.5	None^b^	100.0 ± 3.4	pNP palmitate (C16:0)^b,d^	100.0 ± 2.2
CaCl_2_^b^	100.0 ± 1.2	RbCl	128.4 ± 2.3	pNP myristate (C14:0)	75.4 ± 1.7
CuCl_2_	59.3 ± 0.3	KCl	121.8 ± 4.6	pNP dodecanoate (C12:0)	90.0 ± 4.9
Al_2_(SO_4_)_3_	59.2 ± 0.1	CsCl	104.1 ± 4.7	pNP decanoate (C10:0)	83.2 ± 1.7
CoCl_2_	54.9 ± 0.6	NaCl	94.3 ± 4.4	pNP caproate (C6:0)	29.3 ± 0.7
MnCl_2_	54.9 ± 1.2	LiCl	92.7 ± 2.3	pNP valerate (C5:0)	34.3 ± 2.1
NiCl_2_	53.3 ± 0.6	Ba(Ac)_2_	85.4 ± 5.3	pNP butyrate (C4:0)	25.6 ± 0.8
FeSO_4_	37.4 ± 0.9	CaCl_2_	73.4 ± 4.2	pNP acetate (C2:0)	1.9 ± 0.2
Pb(Ac)_2_	33.9 ± 0.2	SnCl_2_	67.6 ± 3.4	Optimal temperature ^e^	
CdSO_4_	32.7 ± 0.3	NiCl_2_	66.0 ± 1.0	9.9°C	38.4 ± 6.4
HgCl_2_	29.5 ± 0.9	MgCl_2_	64.5 ± 1.9	20.5°C	93.8 ± 6.6
MgCl_2_	4.0 ± 0.1	HgCl_2_	62.3 ± 1.3	30.0°C^b^	100.0 ± 5.1
AgNO_3_	0.9 ± 0.2	CoCl_2_	53.8 ± 0.7	40.0°C	37.7 ± 7.7
Ba(Ac)_2_	0.8 ± 0.1	CuCl_2_	52.4 ± 0.9	50.7°C	14.8 ± 3.5
KCl	< 0.5	CdSO_4_	49.8 ± 2.3	60.6°C	11.1 ± 3.6
ZnCl_2_	< 0.5	FeCl_3_	48.8 ± 5.3	Sodium chloride	
SnCl_2_	< 0.5	MnCl_2_	47.1 ± 1.7	None^b^	100.0 ± 2.9
LiCl	< 0.5	ZnCl_2_	44.5 ± 2.9	0.01 M	109.4 ± 4.5
RbCl	< 0.5	Al_2_(SO_4_)_3_	44.8 ± 1.3	0.05 M	121.8 ± 2.0
CsCl	< 0.5	FeSO_4_	33.4 ± 0.5	0.1 M	126.0 ± 3.2
NaCl	< 0.5	Pb(Ac)_2_	26.9 ± 0.4	0.2 M	148.8 ± 4.1
FeCl_3_	< 0.5	AgNO_3_	8.2 ± 0.4	0.5 M	349.4 ± 26.1
Anion^c ^(1 mM)		Anion^c ^(10 mM)		1.0 M	1346.5 ± 38.2
None^b^	100.0 ± 4.5	None^b^	100.0 ± 1.4	1.5 M	1501.5 ± 74.6
CH_3_COO-	108.4 ± 7.8	SO_4_^2^-	110.4 ± 0.9	2.0 M	1333.5 ± 95.4
NO_3_-	102.6 ± 2.6	SO_3_^2^-	109.1 ± 1.0	2.5 M	37.2 ± 7.5
SO_4_^2^-	99.3 ± 3.4	CH_3_COO-	108.4 ± 2.2	3.0 M	114.3 ± 1.6
Cl -	97.4 ± 5.4	Cl -	104.5 ± 0.8	3.5 M	164.4 ± 8.1
SO_3_^2^-	96.9 ± 3.5	NO_3_-	102.2 ± 2.7	4.0 M	170.9 ± 1.7
PO_4_^3^-	93.2 ± 6.2	PO_4_^3^-	68.0 ± 1.4	Modifying reagent (1 mM)	
Detergent (0.1%)		Detergent (1%)		None^b^	100.0 ± 0.6
None^b^	100.0 ± 3.7	None^b^	100.0 ± 1.2	PMSF	13.1 ± 1.2
CTAB	175.0 ± 3.7	Triton X-100	34.3 ± 0.3	DEPC	35.4 ± 0.4
NLS	130.5 ± 0.1	NP40	29.0 ± 0.5	Chelating agent (10 mM)	
Triton X-100	114.1 ± 3.6	NLS	16.9 ± 0.4	None^b^	100.0 ± 1.5
NP40	102.8 ± 2.6	Tween 40	15.4 ± 0.7	EDTA	50.0 ± 2.4
Tween 20	56.4 ± 1.8	Tween 20	15.4 ± 0.2	EGTA	22.4 ± 0.6
SDS	50.7 ± 2.4	Tween 80	12.9 ± 0.3	Emulsifying agent	
Tween 40	38.6 ± 1.0	CTAB	12.0 ± 0.7	None^b^	100.0 ± 2.2
Tween 80	32.6 ± 0.3	SDS	8.8 ± 0.2	Gum arabic (0.5%)	186.5 ± 1.3

All measurements were performed using the *p*NPP method. Standard errors of the mean values are shown. ^a ^The enzyme was preincubated with 1 mM EDTA in order to chelate any metal bound to the enzyme molecule prior to cation addition. ^b ^Reference compound/condition set as 100% activity. ^c ^All anions from sodium salts to avoid cation interference. ^d ^The specific activity for this compound was 196.5 U/mg. ^e ^Apparent optimum temperature over 1 h reaction time.

#### Effect of anions on the LipC12 activity

The effect of anions on LipC12 activity was also tested. All anions used were added in the form of sodium salts and none showed a significant effect on activity at 1 mM (Table 1). However, PO_4_^3- ^at 10 mM decreased the enzyme activity by about 30%, suggesting that PO_4_^3- ^competes with LipC12 for Ca^2+^.

#### LipC12 enantioselectivity and substrate specificity against *p*NP esters

LipC12 is most active against longer-chain nitrophenyl acyl esters, with relative activities above 75% for even-numbered acyl chain lengths from C10:0 to C16:0 (Table 1). For shorter acyl chain lengths (C2:0 to C6:0), the relative activities are below 35%. This preference for longer chain lengths is typical of a true lipase and not an esterase [1]. In the Quick E test for enantioselectivity, LipC12 gave an S/R rate ratio of 1.53 ± 0.05 with pure glycidyl butyrate enantiomers, indicating that LipC12 is not enantioselective for this compound under the conditions of the experiment. However, this does not eliminate the possibility that LipC12 will show enantioselectivity for other compounds or under other reaction conditions.

#### Effect of detergents, gum arabic and modifying agents on LipC12 activity

Detergents such as Triton X-100 are usually added to lipase substrate emulsions to improve the emulsion quality, making the substrate more accessible. However, depending on the concentration used, detergents can also cause lipases to denature. For LipC12, at 0.1% (v/v) concentration, the detergents CTAB, NLS and Triton X-100 enhanced the activity up to 175% (Table 1). NP40 had no effect and SDS inhibited the activity by about 50%. Although Tween 20, 40 and 60 are non-ionic detergents, as are Triton X-100 and NP40, they inhibited the activity as much or more than SDS. This effect is probably due to the long acyl ester chains of these detergents making them substrates for LipC12 and therefore competitive inhibitors in the assay. Interestingly, Tween 80 was the most effective inhibitor of *p*NPP hydrolysis, suggesting that LipC12 has a preference for esters of C18 fatty acids over C16 (Tween 40) and C12 (Tween 20). At 1% (v/v), all detergents decreased the activity significantly, with LipC12 tolerating best NP40 and Triton X-100. Since gum arabic is often added to stabilize lipase substrate emulsions [44], it is important to know its effect on LipC12 activity. At 0.5% (w/v) the relative activity almost doubled. Modifying agents such as PMSF (specific inhibitor of serine hydrolases) and DEPC (histidine residue modifier) at 1 mM concentrations strongly reduced LipC12 activity (Table 1). This effect suggests that LipC12 does indeed have a catalytic triad containing serine and histidine residues [3].

#### Residual activity of LipC12 after incubation in organic solvents

The activities of LipC12 after 48 h incubation at 4°C in 15% and 30% (v/v) of organic solvents was inhibited only in the cases of 15% (v/v) acetone and 15% (v/v) acetonitrile (Table 2). In all other cases the LipC12 activity after incubation was stimulated. This phenomenon is in fact quite well known. For example, *Candida rugosa *lipase is activated by organic solvents, which keep the lid of the enzyme in the open conformation, facilitating the access of the substrate to the active site [45,46]. Although the three-dimensional structure of LipC12 has not yet been determined, the increased LipC12 activity in this experiment suggests the existence of a lid that is converted from closed to open conformation in the presence of organic solvents. These findings are particularly significant, due to the fact that organic solvents have been used in biodiesel production through biocatalysis.

**Table 2:** Effect of organic solvents, pH, NaCl and temperature on the stability of LipC12

Variable	Residual	Variable	Residual	Variable	Residual
	activity (%)		activity (%)		activity (%)
Solvent stability^a^		Solvent stability^a^		Sodium chloride^d^	
None^b^	100.0 ± 5.7	DMF 15%	223.1 ± 12.5	None^b^	100.0 ± 6.5
Methanol 15%	1204.1 ± 20.6	DMF 30%	355.9 ± 2.8	0.9 M	100.9 ± 8.5
Methanol 30%	1561.1 ± 72.4	Acetonitrile 15%	62.7 ± 1.9	1.9 M	102.2 ± 7.1
Ethanol 15%	587.6 ± 28.2	Acetonitrile 30%	316.2 ± 69.7	2.3 M	99.7 ± 5.0
Ethanol 30%	428.4 ± 39.1	pH stability^c^		2.8 M	109.0 ± 11.7
1-Propanol 15%	329.0 ± 15.0	AC 3	0.0	3.2 M	99.1 ± 7.7
1-Propanol 30%	1161.9 ± 27.4	AC 4	83.9 ± 5.3	3.7 M	111.8 ± 6.8
2-Propanol 15%	170.6 ± 3.3	AC 5	85.7 ± 5.9	Thermostability^e^	
2-Propanol 30%	696.2 ± 57.9	AC 5.5	70.5 ± 4.0	0°C^b^	100.0 ± 4.23
Glycerol 15%	167.8 ± 2.1	MES 5.5	77.9 ± 6.1	20.5°C	100.0 ± 1.0
Glycerol 30%	233.0 ± 6.4	MES 6	92.4 ± 5.3	30.0°C	96.2 ± 7.7
THF 15%	463.6 ± 139.4	MES 7	95.5 ± 1.0	40.0°C	100.0 ± 4.2
THF 30%	1690.9 ± 29.0	HEPES 7	95.7 ± 8.1	50.7°C	94.0 ± 6.4
Acetone 15%	66.5 ± 1.6	HEPES 7.5^b^	100.0 ± 4.9	60.6°C	75.8 ± 4.0
Acetone 30%	624.2 ± 3.5	GLC 7.5	96.9 ± 5.1	70.0°C	64.9 ± 8.3
Dioxane 15%	505.4 ± 22.8	GLC 8	94.8 ± 3.1	81.0°C	31.4 ± 0.7
Dioxane 30%	509.7 ± 47.3	GLC 9	98.4 ± 10.6	90.2°C	6.4 ± 1.3
DMSO 15%	263.7 ± 10.1	GLC 10	99.0 ± 5.3		
DMSO 30%	770.1 ± 41.7	GLC 11	91.5 ± 6.3		

All measurements were performed using the *p*NPP method. Standard errors of the mean values are shown. ^a ^The enzyme was incubated for 48 h at 4°C in this assay. ^b ^Reference compound/condition set as 100% activity. ^c ^LipC12 was incubated for 24 h at 4°C in each pH buffer. ^d ^The enzyme was incubated for 24 h at 4°C in this assay. ^e ^The enzyme was incubated for 1 h at each temperature.

#### Effect of NaCl and KCl concentrations on LipC12 activity and stability

Addition of NaCl to a *p*NPP emulsion until 1.5 M increased LipC12 activity up to 15 fold (Table 1), however, the activity decreased with further increases in the NaCl concentration. Since LipC12 is stable at NaCl concentrations up to 3.7 M (Table 2) the decrease in activity above 1.5 M is probably due to decreased substrate solubility, which was indicated by the emulsion becoming turbid at 3 M NaCl. The same results about LipC12 stability were observed for KCl (results not shown). Enzyme stability at high salt concentrations might indicate that the enzyme will be stable in the low water activity environments that occur in biocatalytic reactions carried out in organic solvents [47].

#### Effect of pH on LipC12 stability

Determination of the pH stability of enzymes is important for identifying nondenaturing pH values of buffers for purification, storage and reaction steps. LipC12 showed a broad pH tolerance, with over 90% residual activity after 24 h incubation at pH values ranging from 6.0 to 11.0 (Table 2). At pH 3 LipC12 lost all activity and in the range of 4.0 to 5.5 the residual activity was between 70 and 90%. The enzyme showed a local stability minimum at pH 5.5, probably caused by isoelectric precipitation [48].

#### Effect of temperature on LipC12 activity and stability

In an enzymatic process there is a critical play between thermostability and the effect of temperature on activity. It is necessary to identify a reaction temperature that at the same time allows a reasonably high rate of reaction and keeps the rate of denaturation at a reasonably low level. LipC12 showed maximum activity at 30°C (Table 1) when incubated for 1 h and enzyme stability (during 1 h incubation) was not affected up to 50°C (Table 2). After 1 h at 70°C the enzyme retained about 65% of its original activity.

### Titrimetric determinations of lipase activity using triacylglycerol substrates

#### LipC12 substrate specificity against triacylglycerols

**Figure 4:**
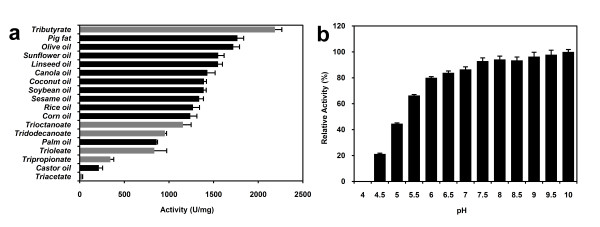
**Enzymatic activity measurements obtained using the titrimetric method with triacylglycerols**. (a) LipC12 substrate specificity against triacylglycerols. Among long-chain triglycerides, LipC12 showed the highest activities for pig fat and olive oil. (b) Effect of pH on LipC12 activity. LipC12 showed activity above pH 4.5 and it is more active under neutral to alkaline conditions.

As shown in Figure 4a, LipC12 was most active against tributyrin (2187 U/mg), pig fat (1767 U/mg) and olive oil (1722 U/mg), with relatively low activities against tripropionin (344 U/mg), castor oil (215 U/mg) and triacetin (28 U/mg). On the other hand, zymogram analysis (Figure 1d) and plate screening tests (results not shown) showed larger hydrolysis halos against trioctanoin than against tributyrin. In addition, when using *p*NP substrates, LipC12 showed a preference for the C16 rather than the C4 acyl group. These results can be explained by the fact that the activity not only depends on the length of the acyl chain, but is also affected by factors such as the presence or absence of insaturations or hydroxyl groups in the acyl chain, the solubility of the triacylglycerol and the quality of the emulsion. LipC12 showed a relatively low activity against castor oil, probably because of the unusual hydroxyl functional group on the twelfth carbon of the ricinoleic acid.

#### Effect of pH on LipC12 activity

The effect of pH on activity was studied using tributyrin, in order to reduce buffering effects in the titrations: tributyrin has the smallest correction factor, due to its lower pK_a_, compared with tricaprylin and triolein. Over a pH range of 4 to 10, LipC12 displayed more than 90% relative activity at pH values above 7; the maximum activity was at pH 10 and no activity was observed below pH 4.5 (Figure 4b). Thus this lipase is more active under neutral and alkaline conditions. Measurements at pH 11 and above were unreliable due to nonenzymatic tributyrin hydrolysis.

## Discussion

The combination of fat contaminated soil and the prokaryotic DNA extraction protocol used were probably responsible for the high hit rate (0.53%) of lipolytic active clones obtained. So far only two other works have reported a hit rate of this magnitude. Recently, Liaw *et al*. (2010) obtained a hit rate of 0.31% (12 positives) for lipolytic clones with a metagenomic library constructed from an activated sludge of a swine wastewater treatment facility. They attributed their high hit rate to the sample source, which caused a natural enrichment in lipolytic organisms. A comparable effect seems to have occurred with the high-fat soil used in this study. Using the prokaryotic DNA extraction protocol, Hårdeman and Sjöling (2007) obtained a hit rate for lipolytic clones of 1% from a metagenomic library of Baltic Sea sediment with more than 7000 fosmids. When the prokaryotic DNA extraction technique or the enrichment step prior to DNA extraction are not applied, the hit rate of lipolytic clones in metagenomic libraries is typically lower, ranging from 0.001 to 0.081% [28].

The prokaryotic DNA extraction procedure circumvents two main problems. First, it is very difficult to extract DNA of high molecular weight for fosmid cloning from a soil with high organic matter content by direct lysis procedures [49]. Second, even though eukaryotic cells correspond to only 0.1% of the total number of cells in environmental samples, they may contribute about 90% of the total DNA due to their larger genome size. This eukaryotic DNA is unlikely to be expressed properly in *E. coli *but increases the library size [50].

In addition to the hit rate, the library size is a critical factor for success in a metagenome project. Metagenomics libraries used have diverse sizes, in most cases varying from few thousands to one million clones [51-53]. In the current work, both the elevated hit rate and the large size of the metagenomic library (approximately 500,000 clones) allowed the detection of 2,661 lipolytic clones, the largest number of lipolytic clones so far reported in a metagenomic study. Moreover, the three-stage screening strategy was able to discriminate triolein-active clones among the tricaprylin- and tributyrin-active clones, enabling the selection of a true lipase as well as the acquisition of lipolytic clone sublibraries for further screenings against oils and fats of interest.

Few lipases with high activity against long-chain triglycerides such as vegetable oils or animal fat have been found so far through the metagenomic approach. To the best of our knowledge, among metagenome-derived lipases, LipC12 has the highest specific activities against long-chain triglycerides (1767 and 1722 U/mg for pig fat and olive oil, respectively). In other metagenomic studies, EML1, a new cold-active lipase from a sediment metagenome, had a preference for medium-chain triglycerides, with a specific activity against trilaurin (C12) of 203 U/mg [35]; the lipases RlipE1 and RlipE2, isolated from a metagenomic library of cow rumen, had specific activities of 346 and 232 U/mg, respectively, against triolein (C18) [36]; and LipG, from a metagenomic library of tidal flat sediments, had a specific activity against *p*NPP of 459 U/mg, while for triglycerides only relative activities were presented, with the highest activity against triolein, followed by tricaprylin and tributyrin [33]. Other metagenome-derived lipases showed hydrolysis halos in triolein or olive oil plate assays but the specific activities against these substrates were not measured [32,34,37].

In fact, the specific activity of LipC12 is comparable to that of the lipases of *Rhizopus oryzae, Rhizomucor miehei *and *Thermomyces lanuginosus *(formerly *Humicola lanuginosa*), which showed activities against olive oil of 1000, 3300 and 2900 U/mg, respectively [54]. These are industrially used lipases with well-known high activity against long-chain triglycerides [4,12,55].

## Conclusions

We obtained a high hit rate of lipolytic activity in our metagenomic study by combining three strategies, namely the use of fat-contaminated soil, the use of a prokaryotic DNA enrichment step and the use of a three-step screening strategy in which the last step tested for true lipase activity. The lipase that we isolated, LipC12, is a novel lipase. Although it was initially selected solely on the basis of its high activity against triolein, it has characteristics that make it suitable for biotechnological applications such as activity and stability over a wide range of pH values, high activity and high thermal stability at ambient temperatures and stability in water-miscible organic solvents and at high salt concentrations. Moreover, production of recombinant LipC12 is convenient due its high solubility, easy purification through affinity chromatography and chaperone-free folding. LipC12 is a promising candidate for improvement of stability and activity by protein engineering, once the LipC12 three-dimensional structure is determined, or by directed evolution.

## Methods

### Bacterial strains and plasmids

*Escherichia coli *EPI300™-T1^R ^and pCC2FOS fosmid vector (CopyControl Fosmid Library Production Kit, Epicentre Biotechnologies, Madison, WI, USA) were used for constructing the metagenomic library. *E. coli *DH10B and the vectors pUC18 and pCR2.1 (Invitrogen Life Technologies, USA) were used for subcloning steps. *E. coli *BL21(DE3) and pET-28a(+) vector (Novagen, Madison, WI, USA) were used as the recombinant protein expression system.

### Chemicals and enzymes

FideliTaq PCR Master Mix (USB, Cleveland, OH, USA) was used for DNA amplification. T4 DNA ligase, T4 DNA polymerase, Klenow fragment, T4 polynucleotide kinase, shrimp alkaline phosphatase (SAP), restriction enzymes and the protein molecular mass marker were purchased from Fermentas (Glen Burnie, MD, USA). The HiTrap Chelating HP column was purchased from GE Healthcare (Uppsala, Sweden). The nitrophenyl ester series, triacetin, tripropionin, tributyrin, trioctanoin, tridodecanoin, triolein, castor oil, BES buffer, (*R*)-glycidyl butyrate, (*S*)-glycidyl butyrate, resorufin butyrate and *p-*nitrophenol were purchased from Sigma-Aldrich (St. Louis, MO, USA). The natural oils for lipase analysis were commercial products purchased from a supermarket. All other chemicals used for lipase analysis were of analytical grade.

### Sample collection and DNA extraction

Soil samples were collected from the banks of an anaerobic lagoon (GPS position, 24°56'11.11"S, 50° 7'27.06"W) of the wastewater treatment plant of a meat packing and dairy industry located in the state of Paraná, Brazil. The average soil temperature was 30°C and soil samples up to 3 cm of soil depth were aseptically stored for 24 h at ambient temperature and subjected to DNA extraction by an indirect lysis method [56], in which prokaryotic cells were separated from the sediment by low-speed centrifugation before cell lysis.

### Metagenomic library construction and screening for lipolytic activity

Purified DNA samples were size-separated on a 0.7% low melting point (LMP) agarose gel in TAE buffer at 20 V/cm. DNA bands from 20 to 40 kb were excised from the gel and extracted by the phenol method [57]. A metagenomic DNA library was constructed using the CopyControl Fosmid Library production kit, according to the manufacturer's protocol (Epicentre Biotechnologies, Madison, WI, USA). For activity screening, the transformed cells were plated onto modified Luria-Bertani (LB) agar plates (5 g/L peptone, 3 g/L yeast extract, 13 g/L bacteriological agar, 10 g/L gum arabic) containing 1% (v/v) emulsified tributyrin as substrate. Cells were grown at 37°C for four days and transformants with clear halos around individual colonies were chosen as possible lipase/esterase producing clones. The selected clones were transferred to 96-well microtiter plates with Terrific Broth and subjected to a second screening for lipolytic activity on the modified LB agar except that 1% (v/v) tricaprylin was used instead of tributyrin. Tricaprylin positive clones were transferred to 96-well microtiter plates and stored. True lipase producing clones were identified amongst the tricaprylin active clones by screening on modified LB agar containing 1% (v/v) triolein. Three clones that showed the strongest lipase activity (FosC12, FosE6 and FosH10) were further analyzed.

### Subcloning and identification of the lipase gene

Sublibraries of the FosC12, FosE6 and FosH10 fosmids were constructed in pUC18 vector and subclones were screened for lipolytic activity on modified LB agar medium with 1% (v/v) triolein. The inserts of active subclones were sequenced on an ABI 377 Automated Sequencer (Applied Biosystems, USA) from both ends using DYEnamic ET Dye Terminator Kit (GE Life Sciences, USA). Sequence assembly and editing were performed with the CodonCode Aligner software (CodonCode Corporation, Dedham, MA, USA). The open reading frames (ORFs) were identified with the ORF Finder tool [58] and the amino acid sequences were compared with the non-redundant sequence database deposited at the NCBI using BLAST [59]. Fosmids FosC12, FosE6 and FosH10 had the same lipase gene. Thus, further cloning steps were performed using the FosC12 fosmid and the lipase protein sequence is hereafter denominated LipC12 (*lipC12 *gene).

### Lipase sequence analysis and phylogenetic tree construction

Prediction of transmembrane regions and signal peptide sequence were performed using the TMHMM 2.0 [60] and SignalP 3.0 servers [61], respectively. Protein solubility prediction was carried out with the system SOSUI [62]. The ProtParam tool was used to calculate the theoretical parameters of the protein [63]. Multiple sequence alignment was performed with the ClustalW algorithm [64] in combination with Geneious software (Biomatters Ltd, Auckland, New Zealand). Phylogenetic analysis was carried out with the neighbor-joining method using MEGA version 5. Bootstrapping (10,000 replicates) was used to estimate the confidence levels of phylogenetic reconstructions [65]. GeneDoc version 2.7 was used for displaying and highlighting the multiple sequence alignments.

### Cloning of *lipC12 *gene

The forward primer LipC12.L (5'CAACGTCAAAGAGGTTATTC3') and the reverse primer LipC12.R (5'CGAGTGCTATCGTTCATTTA3') were used in a PCR reaction (FideliTaq PCR Master Mix, USB, USA) for the amplification of a 966 bp fragment. The amplified gene was first cloned into the pCR 2.1 vector (TA Cloning Kit, Invitrogen, USA) according to the manufacturer's recommendations and recombinant plasmids were transformed into *E. coli *DH10B competent cells by electroporation. White colonies were picked, plasmids were extracted [66] and their inserts were sequenced with M13 forward and reverse primers to confirm the absence of mutations in *lipC12*. Recombinant plasmid was then digested with NdeI (cut at *lipC12 *translation start codon) and BamHI. The insert was ligated into the pET28a(+) vector, which had been previously digested with the same restriction enzymes and dephosphorylated by SAP, yielding plasmid pET28a-*lipC12*, the insert of which was end-sequenced with the T7 promoter and terminator primers. Plasmid pET28a-*lipC12 *was then transformed into *E. coli *BL21(DE3) cells to express the recombinant (His)6-tagged LipC12 lipase.

### Overexpression and purification of recombinant LipC12 lipase

*E. coli *BL21(DE3) cells carrying the pET28a-*lipC12 *plasmid were grown in 200 mL of LB medium at 37°C until an OD600 of 0.5 and induced by the addition of isopropyl-β-D thiogalactopyranoside (IPTG) to a final concentration of 0.5 mM. The induced culture was incubated for a further 16 h at 20°C before harvesting of the cells by centrifugation (10,000 × g for 5 min) at 4°C. The cell pellet was resuspended in 35 mL of lysis buffer (50 mM Tris-HCl pH 8.0, 500 mM NaCl, 10 mM imidazole, 10 mM β-mercaptoethanol, 10% (v/v) glycerol, 0.25% (w/v) Nonidet P-40) and disrupted by ultrasonication in an ice bath (10 cycles of 60 s pulses, 90 W, with 30 s intervals), using a SONICATOR^® ^XL 2020 (Heat systems-Ultrasonics Inc., New Highway, Farmingdale, NY, USA). The crude extract was then centrifuged at 15,000 × g for 30 min at 4°C to pellet the cell debris. The supernatant containing the His-tagged protein was loaded onto a HiTrap Chelating HP column (GE Healthcare, USA), previously loaded with Ni^2+ ^and equilibrated with lysis buffer, using an ÄKTAbasic chromatography system (GE Healthcare, USA). The column was washed with 5 volumes of the lysis buffer and further with 5 volumes of elution buffer (50 mM Tris-HCl pH 8.0, 500 mM NaCl, 10 mM imidazole, 10% (v/v) glycerol). The His-tagged protein was eluted with an increasing gradient of imidazole up to 500 mM in elution buffer. The elution of protein was monitored at 280 nm, protein fractions were analyzed by SDS-PAGE, pooled, dialyzed (50 mM Tris-HCl pH 8.0, 150 mM NaCl, 10 mM CaCl_2_, 50% (v/v) glycerol) and stored at -24°C until use.

### Protein content determination, electrophoresis and zymogram analyses

Protein content was determined using the Pierce BCA Protein Assay Kit (Pierce Biotechnology, Rockford, IL, USA) with bovine serum albumin as the standard. Electrophoresis of protein samples was done with 12% (w/v) SDS-PAGE [67] and the gel was stained with Coomassie Brilliant Blue R-250 and destained with methanol/acetic-acid/water (5/1/4 v/v/v). Densitometric analysis of the stained SDS-PAGE gel was done using LabWorks Image Acquisition and Analysis Software 4.0 (UVP BioImaging Systems, Upland, CA, USA). Lipolytic activity of bands on the SDS-PAGE gel was detected using tributyrin or tricaprylin as substrate [68].

### MALDI-TOF/MS analysis

Matrix-assisted laser desorption/ionization (MALDI) time-of-flight (TOF) mass spectra (MS) were acquired on a MALDI-TOF/TOF Autoflex II spectrometer (Bruker Daltonics, Bremen, Germany) in the reflector positive ion mode with an acceleration voltage of 20 kV, delay time of 150 ns and acquisition mass range 800-3200 Da. Spots were excised manually and in-gel digested with sequencing grade modified trypsin (Promega, Madison, USA) as described elsewhere [69]. The sample was desalted using a ZipTipC_18 _pipette tip (Millipore Corporation, Bedford, MA, USA) and eluted directly onto the MALDI target plate using MALDI matrix (saturated solution of α-cyano-4-hydroxycinnamic acid in 50% (v/v) acetonitrile and 0.1% TFA). Mass profiles were identified by comparing the peptide masses obtained with *in silico *digestion of the His-tagged protein sequence using PeptideCutter and MS-Digest tools [63].

### Spectrophotometric determinations of lipase activity using *p*NP substrates

Enzymatic activities were determined by following the amount of *p-*nitrophenol released from *p-*nitrophenyl ester at 410 nm [70] for at least 30 min at 30°C in a TECAN Infinite Series M200 microplate spectrophotometer (TECAN, Salzburg, Austria). Unless otherwise described, for the standard assay, the substrate solution was made by mixing a stock solution of 20 mM of *p-*nitrophenyl palmitate (*p*NPP) in acetonitrile/isopropanol (1/4 v/v) with an assay buffer containing Tris-HCl pH 7.5, CaCl_2 _and Triton X-100, under agitation in a water bath at 60°C, until the solution became transparent. Then 230 μL of the substrate solution was pipetted into a 96-well microtiter plate and the reaction was initiated by automatic addition (with the TECAN injector module) of 20 μL of the enzyme solution (50 mM Tris-HCl pH 7.5, 1 mM CaCl_2_) to a final concentration of 5 nM. The final volume of the reaction mixture (Tris-HCl 50 mM, pH 7.5, 1 mM CaCl_2_, 0.3% (v/v) Triton X-100, 5 nM enzyme, 1 mM *p*NPP, 4% (v/v) isopropanol, 1% (v/v) acetonitrile) was 250 μL. All experiments were performed in triplicate, the extinction coefficients of *p-*nitrophenol were determined under each reaction condition and the effect of nonenzymatic hydrolysis of substrates was subtracted. One unit of lipase activity was defined as 1 μmol of *p-*nitrophenol produced per minute. Linear regressions to determine initial reaction velocities and standard deviations of means were performed with Calc software from the OpenOffice.org package.

Substrate specificity for nitrophenyl esters was analyzed under standard conditions using *p-*nitrophenyl acetate (C2:0), *p-*nitrophenyl butyrate (C4:0), *p-*nitrophenyl valerate (C5:0), *p-*nitrophenyl caproate (C6:0), *p-*nitrophenyl decanoate (C10:0), *p-*nitrophenyl dodecanoate (C12:0), *p-*nitrophenyl myristate (C14:0) and *p-*nitrophenyl palmitate (C16:0).

The temperature giving maximum substrate conversion over a 1 h reaction time was determined by incubating the enzyme with *p-*nitrophenyl ester (2 nM enzyme, 10 mM *p-*nitrophenyl butyrate, MES 50 mM pH 6.0, 1 mM CaCl_2_, 0.3% (v/v) Triton X-100, 4% (v/v) isopropanol, 1% (v/v) acetonitrile) in a reaction volume of 50 μL at various temperatures within the range of 10°C to 60°C. In relation to the standard assay, *p-*nitrophenyl butyrate was used instead of *p*NPP to minimize substrate solubility variations with reaction temperature and the reaction pH was decreased to minimize substrate autohydrolysis, which occurs at higher temperatures. The reaction was carried out in a 96-well PCR plate on a Thermal Cycler (Eppendorf, Hamburg, Germany) running in gradient mode. After the reaction time, the plate was immediately chilled on ice and the enzymatic reaction was stopped by the addition of 100 μL of 0.1 M HCl. Then 125 μL was transferred to a 96-well microtiter plate, completed to 250 μL with deionized water and the amount of released *p-*nitrophenol was measured at 348 nm instead of 410 nm: the absorption of *p-*nitrophenol decreases at 410 nm as the pH decreases, due to changes in equilibrium between *p-*nitrophenol and *p-*nitrophenoxide, while 348 nm is the pH-independent isosbestic wavelength of *p-*nitrophenoxide and *p-*nitrophenol [71].

The effect of cations and anions on the enzymatic activity of LipC12 was also investigated. For analyses involving cations, the enzyme at 250 nM was previously incubated with 1 mM EDTA/50 mM Tris-HCl pH 7.5 for 1 h in order to remove cations from the enzyme. Then 20 μL of enzyme solution was added to each well of a 96-well microtiter plate, followed by 25 μL of cation solution and 205 μL of substrate solution (with 0.5 mM of EDTA) giving a reaction mixture (Tris-HCl 50 mM pH 7.5, 1 mM of a specific cation, 0.3% (v/v) Triton X-100, 20 nM enzyme, 1 mM *p*NPP, 0.5 mM EDTA, 4% (v/v) isopropanol, 1% (v/v) acetonitrile) that was monitored at 410 nm, as described above. The effect of cations on the enzymatic activity of LipC12 without previous cation depletion was also tested. For analyses involving anions, cation depletion with EDTA was not performed and a standard assay was carried out except that either 1 mM or 10 mM of a specific anion was added (all anions were added in the form of sodium salts).

Similarly, the effects of lipase inhibitors were investigated. Chelating agents such as ethylenediamine tetraacetic acid (EDTA) and ethylene glycol tetraacetic acid (EGTA) and modifying reagents such as phenylmethylsulfonyl fluoride (PMSF) and diethyl pyrocarbonate (DEPC) were used. The effects on LipC12 activity of commonly used detergents such as Tween 20, Tween 40, Tween 80, Triton X-100, Nonidet P-40 (NP40), sodium dodecyl sulfate (SDS), Sarkosyl NL (NLS) and hexadecyltrimethylammonium bromide (CTAB) were also investigated. The effect of gum arabic, commonly used as an emulsion stabilizer, was checked. In these cases, the activity measurements were done using the standard assay, except that the chemicals listed above were added to the wells before the addition of the substrate and enzyme solutions.

Thermostability was determined by measuring the residual activity after incubating 250 nM of the enzyme (50 mM Tris-HCl pH 7.5, 5 mM CaCl_2_) at various temperatures in the range of 0°C to 90°C for 1 h in 200 μL PCR Eppendorf tubes with mineral oil on top to prevent evaporation. The incubation was carried out using a volume of 50 μL on the Thermal Cycler (Eppendorf, Hamburg, Germany) running in gradient mode. After the incubation time, the tubes were chilled on ice and the residual activity was determined at 10 nM of enzyme in the reaction mixture.

Stability in organic solvents was determined by measuring the residual activity after incubation of the enzyme at 500 nM with 15% or 30% (v/v) organic solvents (200 mM Tris-HCl pH 8.0, 5 mM CaCl_2_) at 4°C for 48 h. In order to equalize the solvent concentration before the residual activity measurement, the solvent concentration of each solvent incubated at 15% (v/v) was corrected to 0.3% (v/v) during dilution of the enzyme (the enzyme was diluted 100-fold before performing the residual activity measurement).

Stability in high salt concentrations, up to 3.7 M, was investigated by incubating LipC12 at 785 nM (1 mM CaCl_2_, 50 mM Tris-HCl pH 7.5) for 24 h at 4°C. Several NaCl and KCl concentrations were tested. All salt concentrations were equalized to 37 mM during the 100-fold dilution of the enzyme prior to the residual activity measurement.

The pH stability was tested after incubation of the purified enzyme for 24 h at 4°C in different buffers. For a pH range of 3.0 to 11.0, the buffers used were 50 mM sodium acetate (pH 3.0 to 5.5), 50 mM MES (pH 5.5 to 7.0), 50 mM HEPES (pH 7.0 to 7.5), and 50 mM glycine (pH 7.5 to 11). The enzyme was diluted 100-fold in 50 mM HEPES pH 7.5 for the residual activity measurement under standard assay conditions.

Determination of lipase enantioselectivity was performed using the Quick E method [72-74]. Substrate solutions were prepared using glycidyl butyrate enantiomers and resorufin butyrate was used as the reference substrate. A buffer/indicator solution was prepared by mixing 4-nitrophenol solution (1.2 mL of a 1.8 mM solution in 1.0 mM BES containing 0.33 mM Triton X-100, pH 7.2), BES buffer (3.3 mL of a 1.0 mM solution containing 0.33 mM Triton X-100, pH 7.2), and acetonitrile (65 μL). Substrate solution (35 μL of a 150 mM glycidyl butyrate enantiomer in acetonitrile) and resorufin ester solution (260 μL of 2.0 mM resorufin butyrate in acetonitrile) were added dropwise with continuous vortexing to form a clear emulsion that was stable for at least several hours. This solution was pipetted into a 96-well polystyrene microplate (100 μL/well). Lipase solution (5 μL in 5 mM BES) was added to each well and the microplate was placed in the microplate reader (TECAN Infinite Series M200 microplate spectrophotometer, TECAN, Salzburg, Austria) and shaken for 10 s. Then the decrease in absorbance at 404 nm and the increase in absorbance at 574 nm were followed for 30 min at 25°C. Blanks without enzyme were carried out for each substrate and data were collected in triplicate. Reaction rates for the reference substrate and each enantiomer were calculated using Eqs. (1) and (2), respectively, using the initial linear parts of the curves of absorbance versus time:(1)(2)

where d*A*_574_/d*t *is the absorbance increase (at 574 nm) per minute, Δε_574 _is the difference in extinction coefficients at 574 nm for resorufin and resorufin butyrate (15,100 M^-1 ^cm^-1^),* l *is the path length (0.3064 cm for a 105 μl reaction volume), *V* is the reaction volume in liters, d*A*_404_/d*t *is the absorbance decrease (at 404 nm) per minute and Δε_404 _is the difference in extinction coefficients for the protonated and unprotonated forms of the 4-nitrophenol (17,300 M^-1^ cm^-1^).

The enantioselectivity was calculated from two measurements (one for each glycidyl butyrate enantiomer) as shown in Eq. (3):(3)

where rate_S _represents the rate of hydrolysis of the (*S*)-glycidyl butyrate enantiomer and rate_refS _is the rate of hydrolysis of the reference substrate (resorufin butyrate) in the presence of the (*S*)-enantiomer. Analogous definitions apply for rate_R _and rate_refR _in terms of the (*R*)-enantiomer.

### Titrimetric determinations of lipase activity using triacylglycerol substrates

The substrate emulsions consisted of triacylglycerol 67 mM, gum arabic 3% (w/v), CaCl_2 _2 mM, Tris-HCl 2.5 mM and NaCl 150 mM, dispersed in distilled water [75]. The solution was emulsified with a handheld mixer (400 watts, Royal Philips Electronics) at high speed, initially for 10 min and then for an additional 2 min immediately before use. The free fatty acids released during the reaction were titrated automatically in a Metrohm 718 STAT Titrino potentiometric titrator (Metrohm, Herisau, Switzerland) with 0.05 M NaOH, for 5 min. The reactions were done in a glass vessel thermostated at 30°C containing 20 mL of substrate emulsion and 6.68 μg of purified enzyme added in 200 μL of solution buffer (Tris-HCl 2.5 mM pH 8, CaCl_2 _5 mM). The titration point was set to pH 8.5. All measurements were performed in triplicate and chemical hydrolysis of the substrate was subtracted. One unit (U) of enzymatic activity was defined as the release of 1 μmol of fatty acid per minute.

The pH optimum for LipC12 was determined using tributyrin as a substrate [40]. The substrate emulsion and reaction conditions were the same as described above except that the setpoint pH was set at different values. Corrections were made for autohydrolysis and for the partial dissociation of butanoic acid assuming a pK_a _of 4.57.

### Nucleotide sequence accession number

The LipC12 nucleotide sequence reported here is available in the GenBank database under the accession number JF417979.

## Competing interests

The authors declare that they have no competing interests.

## Authors' contributions

NK, FOP and EMS conceived, supervised and coordinated this study. AG participated in the experimental design, carried out the cloning steps, protein purification, enzyme analyses and interpretation of data and wrote the manuscript. HF and MM-S contributed to metagenomic library construction. GHC and RAM participated in subcloning and sequencing steps. VPM participated in protein expression and purification, and contributed to interpretation of enzyme activity analyses. NK and DAM contributed to the analysis and interpretation of data and to the writing of the manuscript. EMS and MM-S critically revised the manuscript. All authors read and approved the final manuscript.
